# Non-Neonatal Tetanus in Mogadishu, Somalia: Clinical Characteristics, Management, and Outcomes—First Report from a Tetanus Referral Centre

**DOI:** 10.3390/tropicalmed11080208

**Published:** 2026-07-24

**Authors:** Mohamed Mukhtar Mohamed, Rukia Abukar Abdi, Mohamed Ahmed Ali, Mohamed Abdirahman Jama, Rahma Yusuf Haji Mohamud

**Affiliations:** 1 Department of Emergency Medicine, Mogadishu Somali Turkiye Training and Research Hospital, Mogadishu, Somalia; 2 Faculty of Medicine & Surgery, Salaam University, Mogadishu, Somalia; 3 Faculty of Medicine and Surgery, Jazeera University, Mogadishu, Somalia; 4 Faculty of Medicine & Surgery, Al Hayat Medical University, Mogadishu, Somalia; rukia.abakar@gmail.com; 5 Department of Emergency Medicine, Madina Hospital, Mogadishu, Somalia; 6 Faculty of Health Science, University of Somalia (UNISO), Mogadishu, Somalia; cayda124@gmail.com; 7 Adminstration Department, Madina Hospital, Specialist Hospital, Mogadishu, Somalia; burtuqal12@gmail.com; 8 Health Services Department, Yardimeli Specialist Hospital, Mogadishu, Somalia; samiihayusuf@gmail.com

**Keywords:** tetanus, non-neonatal tetanus, clinical characteristics, in-hospital mortality, vaccination status, Somalia

## Abstract

**Background:** Tetanus remains a life-threatening yet preventable disease, with a disproportionate burden in low- and middle-income countries. Data on non-neonatal tetanus in Somalia, particularly from referral centres managing severe cases, are limited. This study aimed to describe the clinical characteristics, management, and outcomes of patients with non-neonatal tetanus in Mogadishu, Somalia. **Methods:** This retrospective observational study was conducted at Madina Hospital between July 2024 and January 2026. All patients with clinically diagnosed non-neonatal tetanus presenting to the Emergency Department were included. Data on demographics, clinical features, management, complications, and outcomes were extracted from medical records. Continuous variables were summarized as mean ± standard deviation or median (interquartile range), while categorical variables were presented as frequencies and percentages. Associations were assessed using Fisher’s exact test, with *p* < 0.05 considered statistically significant. **Results:** A total of 50 patients were included (mean age 22.24 ± 15.49 years; 84.0% male). Most patients were unvaccinated (80.0%), and severe or very severe disease was present in 64.0% of cases. Generalized muscle spasm was the most common presentation (50.0%). Dysautonomia (42.0%) and respiratory failure (18.0%) were the most frequent complications. The overall in-hospital mortality rate was 26.0%, with respiratory failure accounting for 69.2% of deaths. Mortality was significantly associated with disease severity (*p* < 0.001), complications (*p* = 0.006), antibiotic category (*p* = 0.043), and vaccination status (*p* = 0.0265). **Conclusions:** Non-neonatal tetanus in this referral-based setting is associated with high mortality, predominantly affecting young, unvaccinated individuals presenting with advanced disease. Disease severity and complications, particularly respiratory failure, are the main drivers of outcomes. Strengthening adult immunization, improving early recognition and referral, and expanding access to critical care are essential to reduce tetanus-related mortality.

## 1. Introduction

Tetanus is a life-threatening neurological disorder caused by tetanospasmin, a potent neurotoxin produced by Clostridium tetani. The disease is characterized by muscle rigidity, painful spasms, trismus, and autonomic dysfunction, and may present as localized, cephalic, or generalized forms, with generalized tetanus representing the most severe and most common clinical presentation requiring hospitalization [1,2]. Management includes administration of tetanus immunoglobulin, wound care, antimicrobial therapy, sedation, and supportive critical care. Despite these interventions, mortality remains high, ranging from 20% to 55%, particularly in low-resource settings [1,3].

Although the global burden of tetanus has declined substantially over recent decades and the disease is entirely preventable through vaccination, it continues to represent a significant public health burden in low- and middle-income countries (LMICs). Global estimates indicate marked reductions in tetanus-related mortality; however, morbidity and mortality remain disproportionately concentrated in regions with incomplete immunization coverage, delayed access to healthcare, inadequate wound management, limited availability of intensive care and mechanical ventilation, and underlying socioeconomic barriers [3,4,5,6]. Sub-Saharan Africa continues to carry a considerable burden of disease, where environmental exposure, trauma, and constrained emergency and critical care services contribute to poor outcomes [7,8].

The World Health Organization (WHO) recommends a six-dose schedule of tetanus toxoid-containing vaccines, consisting of three primary doses during infancy followed by three booster doses in childhood and adolescence to provide lifelong protection [6]. Somalia has adopted the Expanded Programme on Immunization (EPI), which includes tetanus-containing vaccines during infancy; however, vaccination coverage and booster uptake remain suboptimal because of prolonged conflict, disruptions to health services, and limited access to preventive healthcare. Consequently, many adolescents and adults remain susceptible to tetanus, particularly following traumatic injuries and contaminated wounds.

In Africa, tetanus remains prevalent, with high case fatality rates reported across multiple settings. Mortality in non-neonatal tetanus is strongly influenced by disease severity and access to supportive care. Respiratory failure, laryngospasm, aspiration, and autonomic instability are among the leading causes of death, particularly in settings where mechanical ventilation and close monitoring are limited [7,8,9,10]. Previous studies from Ethiopia, Uganda, Sudan, Nigeria, and other African countries have consistently reported high mortality rates and identified factors associated with poor outcomes, including generalized tetanus, delayed presentation, short incubation period, and complications during hospitalization [9,10,11,12,13,14,15,16].

Similar findings have also been reported in recent studies from Asia and other regions, further confirming the global consistency of these risk factors [17,18,19].

Several studies have additionally highlighted prognostic factors associated with mortality, including advanced disease severity, older age, and the presence of complications, emphasizing the importance of early recognition and timely management in improving outcomes [20,21].

In Somalia, published data on non-neonatal tetanus remain limited. Although two recent studies have described tetanus patients in Mogadishu [22,23], these reports primarily evaluated general hospital cohorts and provided limited information on referral-based management. Madina Hospital functions as the country’s principal tetanus referral centre and the primary distribution site for tetanus immunoglobulin (TIG), receiving patients referred from healthcare facilities across Somalia. Consequently, this study represents the first report specifically describing the clinical characteristics, management practices, and outcomes of non-neonatal tetanus patients managed in a national tetanus referral centre.

Madina Hospital in Mogadishu is a major trauma and emergency referral centre and serves as a key national distribution hub for tetanus immunoglobulin. Consequently, patients with suspected tetanus are frequently referred from healthcare facilities across the country, often presenting with advanced disease. This provides a unique opportunity to evaluate non-neonatal tetanus in a referral-based, high-acuity population that has not been adequately characterized.

Therefore, this study aimed to describe the clinical characteristics, management, and outcomes of patients with non-neonatal tetanus presenting to a referral centre in Mogadishu, Somalia, using a retrospective observational design.

## 2. Materials and Methods

### 2.1. Study Design and Setting

This was a retrospective observational study conducted at Madina Hospital in Mogadishu, Somalia, one of the oldest public hospitals in the country, established in 1968. The hospital is operated under the Somali Police and is widely recognized as a major trauma and emergency referral centre, providing comprehensive emergency and critical care services.

Madina Hospital serves as a key national distribution centre for tetanus immunoglobulin (TIG), supported by the Somali Committee of the Red Cross (ICRC). Patients with suspected or confirmed tetanus are frequently referred from healthcare facilities across the country to receive TIG therapy. Consequently, the hospital manages a high proportion of severe tetanus cases.

The study was conducted over an 18-month period from July 2024 to January 2026.

### 2.2. Study Population

All patients with non-neonatal tetanus presenting to the Emergency Department during the study period were eligible for inclusion. Patients were consecutively included to minimize selection bias.

Inclusion criteria were patients of any age (excluding neonates) admitted with a clinical diagnosis of tetanus. Exclusion criteria included alternative diagnoses causing muscle rigidity, incomplete clinical data, and patients who were transferred, discharged early, or left against medical advice before completion of initial evaluation.

Vaccination status was obtained from patients, relatives, vaccination cards when available, and documented medical records. Patients were classified as vaccinated (completed primary tetanus vaccination), partially vaccinated (incomplete primary vaccination or uncertain booster history), or unvaccinated (no previous tetanus vaccination).

### 2.3. Diagnosis of Tetanus

Tetanus was diagnosed clinically based on characteristic features, including trismus, generalized muscle rigidity, and painful muscle spasms, with or without dysphagia or autonomic dysfunction. Laboratory confirmation was not required, in accordance with standard clinical practice.

Disease severity was classified using the Ablett classification. Mild (Grade I) included mild rigidity without respiratory compromise; moderate (Grade II) included moderate spasms without respiratory failure; severe (Grade III) comprised severe spasms with respiratory involvement; and very severe (Grade IV) included severe disease with autonomic dysfunction.

### 2.4. Data Collection

Data were collected retrospectively from medical records using a standardized structured data collection form. Variables recorded included demographic characteristics (age and sex), clinical features (presenting symptoms, portal of entry, disease severity, and vaccination status where available), management interventions (tetanus immunoglobulin administration and dose, antibiotic therapy, sedation, mechanical ventilation, and ICU admission), and clinical outcomes (complications, length of hospital stay, and in-hospital mortality).

### 2.5. Outcome Measures

The primary outcome measure was in-hospital mortality. Secondary outcomes included ICU admission, in-hospital complications, and length of hospital stay.

### 2.6. Ethical Approval

Ethical approval was obtained from the institutional review board of Madina Hospital. The requirement for informed consent was waived due to the retrospective nature of the study. The study was conducted in accordance with the principles of the Declaration of Helsinki, and patient confidentiality was strictly maintained.

### 2.7. Statistical Analysis

All data were recorded and analyzed using SPSS version 25.0 (IBM SPSS Statistics, Armonk, NY, USA: IBM Corp.). Continuous variables were expressed as mean ± standard deviation, while categorical variables were presented as frequencies and percentages. Normality of data distribution was assessed using the Kolmogorov–Smirnov test and histogram analysis. The categorical variables were analyzed using Fisher’s exact test due to small, expected cell counts. A *p*-value < 0.05 was considered statistically significant.

## 3. Results

### Clinical Features and Outcomes of Tetanus: A Retrospective Study

Among 50 patients with tetanus, the mean age was 22.24 ± 15.49 years and 84.0% were male. The most affected age group was 15–29 years (40.0%), and most cases were diagnosed in 2025 (58.0%). Normal vital signs at presentation were observed in 76.0% of patients, while generalized muscle spasm was the most common presenting clinical feature (50.0%). Severe and very severe tetanus accounted for 40.0% and 24.0% of cases, respectively. Most patients had no comorbidity (98.0%) and were admitted to the tetanus ward (78.0%) Table 1.

**Table 1:** Demographic and clinical characteristics of patients with tetanus (n = 50).

Variable	Category	n (%)/Summary
**Age (years)**	Mean ± SD	22.24 ± 15.49
	Median (IQR)	19.5 (21)
	Range	3–75
**Age group**	<15 years	16 (32.0)
	15–29 years	20 (40.0)
	30–44 years	8 (16.0)
	≥45 years	6 (12.0)
**Sex**	Male	42 (84.0)
	Female	8 (16.0)
**Vaccination Status**	Vaccinated	2 (4.0)
	Partially vaccinated	8 (16.0)
	Un-vaccinated	40 (80.0)
**Vital sign on presentation**	Normal	38 (76.0)
	Tachycardia	8 (16.0)
	Hypotension	1 (2.0)
	Hypertension	1 (2.0)
	Tachypnea	1 (2.0)
	Combined hypertension and bradycardia	1 (2.0)
**Risk factor**	Open wound or cut	15 (30.0)
	Nail/sharp object	13 (26.0)
	Unknown	9 (18.0)
	Infected wound	7 (14.0)
	Otitis media	3 (6.0)
	Surgical procedure	1 (2.0)
	Subungual hematoma	1 (2.0)
	Circumcision	1 (2.0)
**Clinical features on presentation**	Generalized muscle spasm	25 (50.0)
	All features	19 (38.0)
	Jaw stiffness (lockjaw)	6 (12.0)
**Severity of illness**	Mild	2 (4.0)
	Moderate	16 (32.0)
	Severe	20 (40.0)
	Very severe	12 (24.0)
**Any comorbidity**	No	49 (98.0)
	Yes	1 (2.0)
**Admission area**	Tetanus ward	39 (78.0)
	General ward	6 (12.0)
	HDU	5 (10.0)

These findings demonstrate that tetanus frequently required supportive inpatient care and was commonly complicated by autonomic dysfunction and respiratory failure.

Table 2 describes how patients were managed during hospitalization and what complications occurred. More than half of patients received combined antibiotics (56.0%), while metronidazole alone and penicillin alone were used in smaller proportions. Dysautonomia management was rarely documented as a separate intervention (4.0%). Regarding hospital procedures, 32.0% required an NGT tube, 20.0% underwent wound debridement, and 4.0% had tracheostomy, while 44.0% required no recorded procedure. Most patients received 3000 IU of immunoglobulin (54.0%), which appears to be the common standard dose in this setting. Hospital stay was generally short to moderate, with 34.0% staying 1–3 days and 32.0% staying 4–7 days; only 14.0% stayed 15 days or more. Complications were common, with dysautonomia being the most frequent complication (42.0%), followed by respiratory failure (18.0%) and acute kidney injury (8.0%). Only 24.0% had no complication. This table indicates that tetanus in this cohort often required supportive inpatient care and was frequently complicated by autonomic and respiratory problems.

Table 3 summarizes the hospital outcomes. More than half of the patients were discharged alive (56.0%), while 4.0% were transferred and 14.0% left against medical advice. The overall mortality rate was 26.0%, as 13 of the 50 patients died. Short-term outcome data confirm that 74.0% were alive and 26.0% died. Among those who died, respiratory failure was the leading cause of death, accounting for 69.2% of deaths, followed by dysautonomia (23.1%) and acute kidney injury (7.7%). This table shows that tetanus in this setting remains associated with substantial mortality, and that deaths were driven mainly by respiratory complications and autonomic instability.

These findings demonstrate that tetanus remained associated with substantial in-hospital mortality, with respiratory failure accounting for most deaths.

Table 4 presents factors associated with mortality. On bivariate analysis, severity of illness, antibiotic category, and complications were significantly associated with mortality. Severity had the strongest relationship with outcome: deaths were concentrated among patients with very severe disease, where 9 of 12 patients died, and the association was highly significant (*p* < 0.001). Complications were also strongly associated with mortality (*p* = 0.006), particularly respiratory failure, which was much more frequent among patients who died than among survivors. Antibiotic category was statistically associated with mortality (*p* = 0.043), with most deaths occurring among patients who received combined antibiotics; however, this may reflect that sicker patients were more likely to receive broader or multiple antibiotics, rather than proving that antibiotics themselves caused death. Other factors, including sex, age group, vital signs, risk factor, clinical features, admission area, dysautonomia management, and procedures, were not significantly associated with mortality. Overall, Table 4 suggests that disease severity and development of complications are the main drivers of poor outcome in this cohort.

## 4. Discussion

This study provides important insights into the clinical characteristics, management, and outcomes of non-neonatal tetanus in a referral centre in Mogadishu, Somalia. The findings demonstrate that tetanus remains a significant cause of morbidity and mortality, particularly among young, predominantly unvaccinated individuals presenting with advanced disease and severe clinical manifestations.

The WHO recommends completion of a six-dose tetanus vaccination schedule during infancy, childhood, and adolescence to ensure lifelong protection [6]. However, booster vaccination remains inadequately implemented in many low-resource settings. Strengthening routine childhood immunization while ensuring completion of booster doses throughout adolescence would substantially reduce the future burden of non-neonatal tetanus.

Our study found a statistically significant association between vaccination status and mortality in tetanus patients, with unvaccinated individuals showing the highest mortality rate. This aligns with previous research that has demonstrated significantly higher mortality in unvaccinated populations. Studies have shown that complete vaccination significantly reduces mortality from tetanus, with unvaccinated individuals facing a case fatality rate as high as 50% in some low-resource settings [1,2]. In contrast, individuals with adequate immunization have much lower mortality rates, highlighting the protective effect of tetanus toxoid vaccination. These findings underscore the importance of increasing vaccination coverage, particularly in areas with low immunization rates, to reduce tetanus-related mortality.

The demographic profile observed in this study, with a predominance of young males (84.0%) and a mean age of 22.2 years, is consistent with previous reports from low- and middle-income countries, where tetanus disproportionately affects economically active age groups and males due to higher exposure to trauma and occupational risks [1,2]. Similar patterns have been reported in studies from sub-Saharan Africa and South Asia, where male predominance often exceeds 70% [3,4].

The high proportion of cases among individuals aged 15–29 years (40.0%) further underscores gaps in adult immunization coverage. Unlike neonatal tetanus, which has declined globally, adult tetanus persists in settings with inadequate booster vaccination programs [5]. This suggests that strengthening routine immunization beyond childhood remains critical in Somalia and similar contexts.

Clinically, most patients presented with generalized muscle spasms (50.0%), and a large proportion had severe or very severe disease (64.0%). These findings align with established evidence that generalized tetanus is the most common and severe form of the disease [6]. The high burden of severe disease in this cohort likely reflects delayed presentation, limited access to early care, and low vaccination coverage, all of which have been described in resource-limited settings [7].

The overall mortality rate of 26.0% observed in this study is comparable to reports from other low-resource settings, where mortality rates range from 20% to over 50% depending on the availability of intensive care support [3,8,12,13,14,15,16]. Although lower than some historical reports, this level of mortality remains unacceptably high and highlights ongoing challenges in the management of tetanus.

The persistence of high mortality in Somalia likely reflects delayed referral, limited availability of intensive care beds, restricted access to mechanical ventilation, and variable availability of tetanus immunoglobulin. Similar healthcare system challenges have been described in Ethiopia, Uganda, Sudan, and Nigeria, where mortality remains strongly associated with disease severity and limitations in critical care resources [9,10,11,12,13,14,15,16].

In the present study, disease severity was the strongest predictor of mortality (*p* < 0.001), with deaths concentrated among patients with very severe disease. This finding is consistent with prior studies demonstrating that severity grading is a key determinant of outcome in tetanus [9,10,20,21]. Severe disease is often associated with complications such as autonomic dysfunction and respiratory failure, which significantly increase the risk of death.

Complications were also significantly associated with mortality (*p* = 0.006), particularly respiratory failure, which was the leading cause of death (69.2%). This is in agreement with existing literature, where respiratory compromise is recognized as the primary cause of death in tetanus patients [6,11]. The need for ventilatory support is a major determinant of survival, and limited access to mechanical ventilation in low-resource settings contributes to higher mortality [12].

The association between antibiotic category and mortality (*p* = 0.043) observed in this study should be interpreted with caution. The higher mortality among patients receiving combined antibiotics likely reflects confounding by indication, where more severely ill patients are more likely to receive broader-spectrum or multiple antibiotic therapies. Similar observations have been reported in previous studies, where treatment patterns often reflect disease severity rather than causality [13].

From a management perspective, variations in tetanus immunoglobulin dosing were observed, primarily due to differences in availability at the referral centre. While most patients received 3000 IU, other doses were administered depending on supply constraints. This reflects real-world challenges in delivering standardized care in resource-limited settings and is consistent with variability reported in other cohorts [8,17,18,19].

Other factors, including sex, age group, vital signs at presentation, risk factors, and procedures, were not significantly associated with mortality. This may be due to the relatively small sample size, which limits statistical power to detect associations. Nonetheless, the findings suggest that clinical severity and complications are the primary drivers of outcome in this cohort.

These findings have important clinical and public health implications. Strengthening adult immunization programs, including booster doses, is essential to reduce disease incidence. Early recognition and timely referral of tetanus cases may reduce disease severity at presentation. In addition, improving access to critical care services, particularly ventilatory support, is crucial to reducing mortality in resource-limited settings.

In conclusion, this study demonstrates that non-neonatal tetanus remains a substantial and preventable cause of morbidity and mortality in this referral-based setting in Somalia. The high proportion of severe disease at presentation, coupled with low vaccination coverage and frequent complications—particularly respiratory failure and dysautonomia—highlights persistent gaps in both preventive and clinical care. Variability in treatment practices, including tetanus immunoglobulin dosing driven by resource availability, further underscores the challenges of delivering standardized management in resource-limited environments. Collectively, these findings emphasize the urgent need to strengthen adult immunization programs, promote earlier recognition and timely referral, and expand access to critical care services to improve outcomes in similar settings.

## 5. Limitations

This was a single-centre study conducted at a referral hospital, which may limit generalizability and introduce selection bias toward more severe cases. The relatively small sample size reduced statistical power, and some variables—including detailed vaccination history and time from injury to symptom onset—were not consistently available. Long-term outcomes after discharge were not assessed. In addition, variations in tetanus immunoglobulin dosing were observed, primarily due to differences in availability at the referral centre, which limited the ability to evaluate standardized treatment effects.

Furthermore, because only 13 deaths occurred during the study period, multivariable logistic regression was not performed to avoid model overfitting and unstable estimates. Therefore, only bivariate analyses were conducted.

## 6. Conclusions

Non-neonatal tetanus remains a significant yet preventable cause of morbidity and mortality in this referral-based setting in Mogadishu, Somalia, predominantly affecting young, unvaccinated individuals presenting with advanced disease. Mortality was primarily associated with severe disease and complications, particularly respiratory failure, highlighting persistent challenges in timely referral and access to critical care. Variability in tetanus immunoglobulin dosing due to resource constraints further reflects the difficulties of delivering standardized care in low-resource settings. Strengthening routine childhood immunization through implementation of the WHO-recommended six-dose tetanus vaccination schedule, ensuring tetanus toxoid vaccination following recovery, promoting booster vaccination among high-risk populations, improving early recognition and referral, and expanding critical care capacity are essential to reduce tetanus-related mortality in Somalia and similar resource-limited settings.

## Figures and Tables

**Table 2:** Treatment, in-hospital management, and complications of patients with tetanus (N = 50).

Variable	Category	n (%)
**Antibiotics**	Metronidazole	11 (22.0)
	Penicillin	7 (14.0)
	Combined	28 (56.0)
	Other	4 (8.0)
**Dysautonomia management**	No	48 (96.0)
	Yes	2 (4.0)
**Procedures done in hospital**	Tracheostomy	2 (4.0)
	NGT tube	16 (32.0)
	Wound debridement	10 (20.0)
	None	22 (44.0)
**Dose of immunoglobulin**	<1000 IU	13 (26.0)
	1000–2999 IU	5 (10.0)
	3000 IU	27 (54.0)
	>3000 IU	5 (10.0)
**Duration of hospital stay**	1–3 days	17 (34.0)
	4–7 days	16 (32.0)
	8–14 days	10 (20.0)
	15+ days	7 (14.0)
**Complications**	Respiratory failure	9 (18.0)
	Acute kidney injury	4 (8.0)
	Electrolyte imbalance	1 (2.0)
	Dysautonomia	21 (42.0)
	Sepsis	3 (6.0)
	None	12 (24.0)

NGT, nasogastric tube; IU, international units. Data are presented as n (%).

**Table 3:** Outcomes and disposition of patients with tetanus (N = 50).

Variable	Category	n (%)
**Disposition**	Discharged	28 (56.0)
	Transferred	2 (4.0)
	Died	13 (26.0)
	Left against medical advice	7 (14.0)
**Short-term outcome**	Alive	37 (74.0)
	Died	13 (26.0)
**Causes of death among patients who died (n = 13)**		
**Cause of death**		**n (%)**
Respiratory failure		9 (69.2)
Acute kidney injury		1 (7.7)
Dysautonomia		3 (23.1)

Percentages for causes of death are calculated among patients who died.

**Table 4:** Factors associated with mortality among patients with tetanus (N = 50).

Variable	Alive (n = 37), n (%)	Died (n = 13), n (%)	*p*-Value
Sex			0.413
Male	32 (86.5)	10 (76.9)	
Female	5 (13.5)	3 (23.1)	
Age group			0.079
<15 years	15 (40.5)	1 (7.7)	
15–29 years	14 (37.8)	6 (46.2)	
30–44 years	4 (10.8)	4 (30.8)	
≥45 years	4 (10.8)	2 (15.4)	
Vaccination Status			0.0265
Vaccinated	2 (4.0%)	0	
Partially vaccinated	4 (8.0%)	4 (8.0%	
Unvaccinated	31 (62.0%)	9 (18.0)	
Vital sign on presentation			0.905
Hypotension	1 (2.7)	0 (0.0)	
Hypertension	1 (2.7)	0 (0.0)	
Tachycardia	5 (13.5)	3 (23.1)	
Tachypnea	1 (2.7)	0 (0.0)	
Combined hypertension and bradycardia	1 (2.7)	0 (0.0)	
Normal	28 (75.7)	10 (76.9)	
Risk factor			0.959
Open wound or cut	10 (27.0)	5 (38.5)	
Surgical procedure	1 (2.7)	0 (0.0)	
Nail/sharp object injury	10 (27.0)	3 (23.1)	
Unknown	6 (16.2)	3 (23.1)	
Otitis media	3 (8.1)	0 (0.0)	
Infected wound	5 (13.5)	2 (15.4)	
Subungual hematoma	1 (2.7)	0 (0.0)	
Circumcision	1 (2.7)	0 (0.0)	
Clinical features on presentation			0.138
Jaw stiffness/lockjaw	5 (13.5)	1 (7.7)	
Generalized muscle spasm	21 (56.8)	4 (30.8)	
All features	11 (29.7)	8 (61.5)	
Severity of illness			<0.001
Mild	2 (5.4)	0 (0.0)	
Moderate	15 (40.5)	1 (7.7)	
Severe	17 (45.9)	3 (23.1)	
Very severe	3 (8.1)	9 (69.2)	
Admission area			0.275
Tetanus ward	28 (75.7)	11 (84.6)	
General ward	6 (16.2)	0 (0.0)	
HDU	3 (8.1)	2 (15.4)	
Antibiotics			0.043
Metronidazole	11 (29.7)	0 (0.0)	
Penicillin	6 (16.2)	1 (7.7)	
Combined antibiotics	18 (48.6)	10 (76.9)	
Other	2 (5.4)	2 (15.4)	
Dysautonomia management			0.456
No	36 (97.3)	12 (92.3)	
Yes	1 (2.7)	1 (7.7)	
Procedures done in hospital			0.551
Tracheostomy	1 (2.7)	1 (7.7)	
NGT tube	11 (29.7)	5 (38.5)	
Wound debridement	7 (18.9)	3 (23.1)	
None	18 (48.6)	4 (30.8)	
Complications			0.006
Respiratory failure	3 (8.1)	6 (46.2)	
Acute kidney injury	2 (5.4)	2 (15.4)	
Electrolyte imbalance	1 (2.7)	0 (0.0)	
Dysautonomia	16 (43.2)	5 (38.5)	
Sepsis	3 (8.1)	0 (0.0)	
None	12 (32.4)	0 (0.0)	

Data are presented as n (%), where percentages are column percentages calculated within outcome groups. *p*-values were calculated using Fisher’s exact test due to small expected cell counts. NGT, nasogastric tube; HDU, high-dependency unit.

## Data Availability

The datasets generated and/or analyzed during the current study are available from the corresponding author on reasonable request.

## Abbreviations

AKI	Acute Kidney Injury
ED	Emergency Department
HDU	High-Dependency Unit
ICU	Intensive Care Unit
ICRC	International Committee of the Red Cross
IQR	Interquartile Range
IU	International Units
LMICs	Low- and Middle-Income Countries
NGT	Nasogastric Tube
SD	Standard Deviation
TIG	Tetanus Immunoglobulin
